# Applying an intersectionality lens to examine health for vulnerable individuals following devolution in Kenya

**DOI:** 10.1186/s12939-019-0917-2

**Published:** 2019-01-30

**Authors:** Rosalind McCollum, Miriam Taegtmeyer, Lilian Otiso, Rachel Tolhurst, Maryline Mireku, Tim Martineau, Robinson Karuga, Sally Theobald

**Affiliations:** 1 Department of International Public Health, Liverpool School of Tropical Medicine, Pembroke Place, Liverpool, UK; 2 LVCT Health, Nairobi, Kenya

**Keywords:** Devolution, Kenya, Intersectionality, Equity

## Abstract

**Background:**

Power imbalances are a key driver of avoidable, unfair and unjust differences in health. Devolution shifts the balance of power in health systems. Intersectionality approaches can provide a ‘lens’ for analysing how power relations contribute to complex and multiple forms of health advantage and disadvantage. These approaches have not to date been widely used to analyse health systems reforms. While the stated objectives of devolution often include improved equity, efficiency and community participation, past evidence demonstrates that that there is a need to create space and capacity for people to transform existing power relations these within specific contexts.

**Methods:**

We carried out a qualitative study between March 2015 and April 2016, involving 269 key informant and in-depth interviews from across the health system in ten counties, 14 focus group discussions with community members in two of these counties and photovoice participatory research with nine young people. We adopted an intersectionality lens to reveal how power relations intersect to produce vulnerabilities for specific groups in specific contexts, and to identify examples of the tacit knowledge about these vulnerabilities held by priority-setting stakeholders, in the wake of the introduction of devolution reforms in Kenya.

**Results:**

Our study identified a range of ways in which longstanding social forces and discriminations limit the power and agency individuals can exercise, but are mediated by their unique circumstances at a given point in their life. These are the social determinants of health, influencing an individual’s exposure to risk of ill health from their living environment, their work, or their social context, including social norms relating to their gender, age, geographical residence or socio-economic status. While a range of policy measures have been introduced to encourage participation by typically ‘unheard voices’, devolution processes have yet to adequately challenge the social norms, and intersecting power relations which contribute to discrimination and marginalisation.

**Conclusions:**

If key actors in devolved decision-making structures are to ensure progress towards universal health coverage, there is need for intersectoral policy action to address social determinants, promote equity and identify ways to challenge and shift power imbalances in priority-setting processes.

## Introduction

Kenya has made remarkable progress towards reducing mortality rates and improving coverage of health services [1]. Despite Kenya’s successes, considerable inequities in health outcomes and uptake of health services exist, disadvantaging individuals who are most vulnerable (ibid). Health inequities go far beyond health service provision, as a consequence of societal privilege and disadvantage, influencing the many aspects of a person’s social location to shape their health and their power to demand and use quality health care [2]. We define power as “the degree of control over material, human, intellectual and financial resources exercised by different sections of society” (page41 [3]). This degree of control and agency influences a person’s opportunity to achieve their highest attainable level of health. Power imbalances are therefore a key driver of avoidable, unfair and unjust differences in health [4]. Social forces, which include socioeconomic and political context, governance, policy and cultural and societal values and norms, influence a person’s social location within their household, community and the wider health system [5]. A person’s social location determines the distribution of power and is influenced by a number of domains, such as race; occupation; gender; location; religion; education; wealth; social capital; disability; age; sexual orientation and other factors. By nature of their ability to shape a person’s material circumstances, social connectedness, psychosocial factors and behaviours, these domains influence their exposure and vulnerability to ‘health affecting factors’ known as the social determinants of health [6]. Together this can give rise to inequitable distribution of health, wellbeing and disease across social groups, as well as access and use of effective health services.

**Fig. 1:**
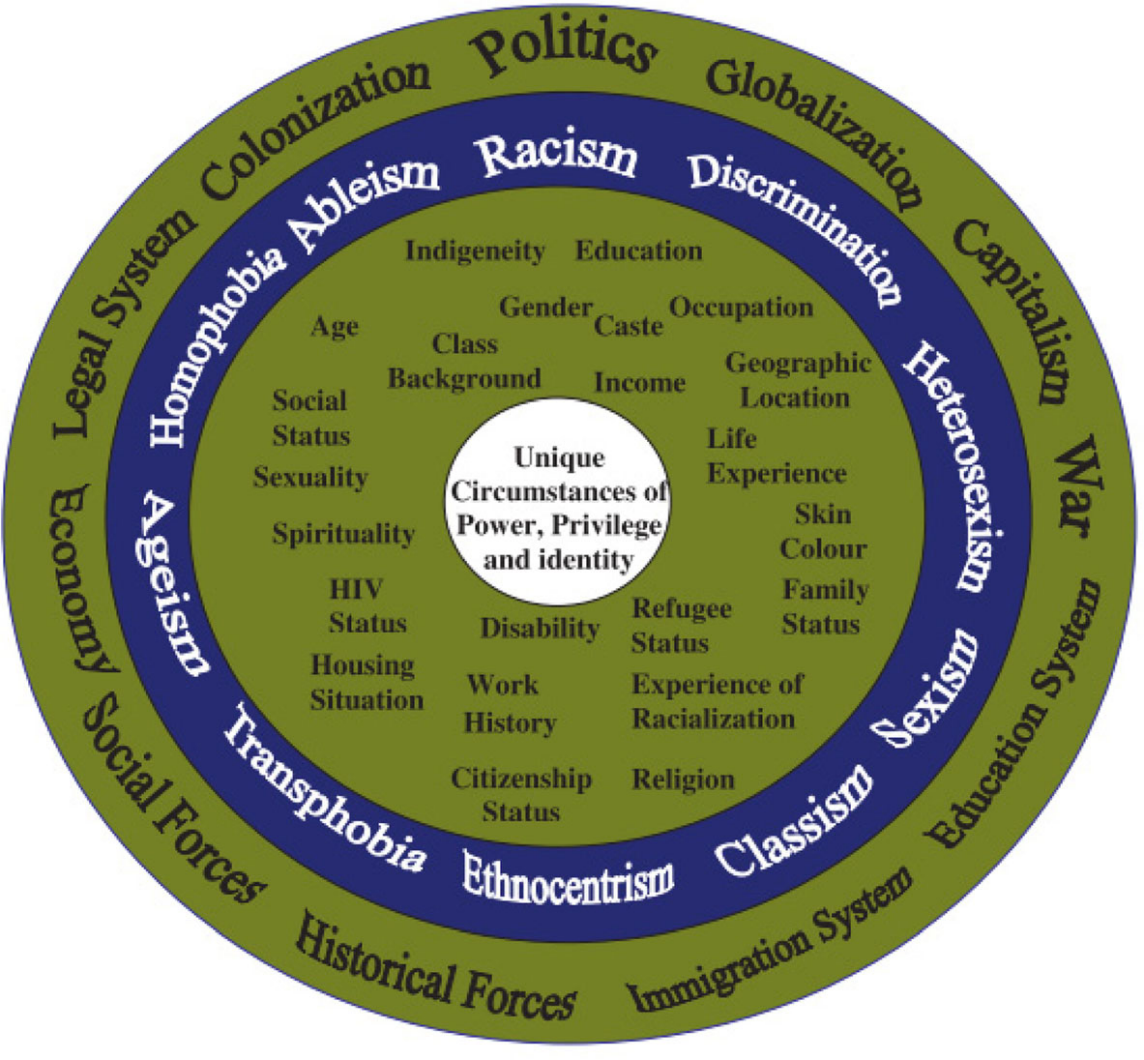
Intersectionality wheel. Source Simpson [8]

Intersectionality allows us to better understand inequality through reflection on the complexity of the world, by reflecting how social domains, e.g. age, gender, are mutually constituted and intersect [7]. It is the concept that, different social domains intersect to produce varying levels of power and privilege for individuals. Intersectionality is also a set of principles regarding the focus and process of research itself (see Table 1). The intersectionality wheel described by Simpson (2009) provides a helpful tool when adopting intersectionality as an approach (see Fig. 1). ‘Societal forces’ (e.g. patriarchy) (outer circle) are expressed through types of discrimination (e.g. sexism) (third wheel from centre) that affect individuals according to their personal social dimensions (e.g. gender) (second wheel from centre) in ways that intersect for each individual in their unique circumstances and specific social location (innermost circle) [8]. Intersectionality approaches have recently been gaining leverage in international health as a way to analyse and address the interplays between different vulnerabilities by trying to uncover underlying power structures that create them [2].

**Table 1:** Intersectionality principles applied within this study

Power, politics, history and social determinants	
• Consideration of how power influences priority-setting, including the processes and systems of power, resulting from the historical, social and political context within which priority-setting takes place.	
• The importance of time and space in considering how historical factors have changed over time leading up to the present day and how positions of privilege or disadvantage have changed since devolution came about.	
• How intersecting social determinants of health (such as gender, place of residence, poverty level) contribute towards ability to engage with priority-setting and to access and use effective health services.	
Analysis approaches	
• Multiple levels of analysis (across national, county, sub-county, health facility and community) to understand how priority-setting has influenced health system performance for community health.	
• Including voices from those not typically heard during priority-setting processes, such as youth from Korogocho informal settlement, Nairobi County.	
• Acknowledgment of our role as researchers, including the power and relationships we bring to the study through applying a reflexivity lens to make explicit our influence as researchers on the choices and decisions made about the methods selected, data collected and analysis conducted as a result of our backgrounds.	

Adapted from [55]

In this paper, we consider power and health as relating to health entitlement and the ways in which a person’s power, or lack thereof in specific contexts and situations, influences the social determinants of their health, including their ability to participate in decisions about health and to access and use quality health services. Kenya’s health inequities are rooted in the historical and social structural forces originating from colonisation, which have contributed to widely varied levels of poverty, education, development, resource allocation and investment for infrastructure and human resources [1, 9–11]. This has resulted in limited availability of the needed resources for effective health service delivery in former marginalised areas (ibid). As a response to growing citizen frustration caused by such wide inequities and inefficiencies of the former centralised government, and post-election violence in 2007/2008, Kenya devolved services (including health) from one central government to 47 county governments [12]. Decentralisation is a dynamic process that transfers authorities or powers for decision making, planning and management of public services from national to subnational levels [13]. There are four main types of decentralisation: de-concentration (shifting authority for administrative functions to sub-national offices within the Ministry of Health); delegation (granting semi-autonomous agencies new administrative powers); devolution (shifting administrative, political and fiscal responsibilities to the sub-national level of a locally elected government) and privatisation (granting ownership to private bodies) [14, 15].

Central to achieving key devolution objectives around tackling “entrenched disparities between regions” (page2 [16]) Kenya has introduced changes to resource allocation between regions through the equitable share fund, which takes into account each county’s poverty level, along with the equalisation fund for formerly marginalised counties [17]. Since devolution was introduced in 2013 in Kenya, the sub-national county authorities have responsibility to identify priorities for health and other services in a participatory manner. According to the new Constitution and the new annual planning and budgeting process at county level [18], county government authorities should hold three public participation meetings to identify citizen needs and priorities and validate whether needs included in draft plans concur with those identified by citizens. Representatives for women, youth and people with disabilities should be invited and given opportunity to participate during community meetings [17]. In addition, citizen representatives should continue to be involved during community and health facility management committee meetings, community dialogue day meetings (community level meetings facilitated by CHWs to analyse community level data) and quarterly health planning meetings. However, recent studies carried out following introduction of devolution have revealed limited community or stakeholder involvement in the process [19], with negative stereotypes towards women with disabilities contributing to their exclusion from public participation meetings [20].

Kenya has introduced measures to reduce financial barriers to using health services, by removing user fees and introducing free maternal health care in 2013, although challenges with disbursement of funds to health facilities following devolution was found to contribute to continued charging of user fees [21]. If Kenya is to meaningfully address disparities and to attain Universal Health Coverage (UHC), then county governments need to pay further attention to how resources are used within each county. This will mean county decision-making actors need to find and use context-appropriate ways to extend quality services to all citizens, particularly individuals who are most vulnerable, to reduce out-of-pocket payments and address the cultural, religious and social barriers which lock people out of effective health coverage [22]. Social forces, which are reinforced by stigma and discriminations must be tackled and county governments will need to go beyond service provision, to address social determinants of health and to empower individuals to overcome the unique barriers they experience to using health services. In order to create insight for county level priority-setting actors, we present findings from a multi-method qualitative study adopting an intersectionality lens to explore power and health following devolution reforms in Kenya. Our paper seeks to reveal how power relations intersect to produce vulnerabilities for specific groups in specific contexts, and to identify examples of the tacit knowledge about these vulnerabilities held by leading priority-setting stakeholders.

## Methods

### Theory and practice

We used a naturalistic approach to explore priority-setting for health and perceptions of equity since devolution. Multiple qualitative methods including key informant interviews, in-depth interviews, focus group discussions and photovoice participatory research captured a range of perspectives from national to community level, including voices from those not normally heard within decision-making processes in the health sector. In acknowledgement of the multiple drivers of inequality in the lives of study respondents and the changing nature of devolution’s implications over time, a number of intersectionality principles were applied to our study to engage with the complexity of the political, social and economic context within which devolution in Kenya is taking place [23] (see Table 1).

Within our study we consider the implications of a number of social dimensions for an individual’s experience of power. Given the dynamic and relational nature of power, exercised through the social, intellectual, economic and political relations between individuals and groups, power can change over time [3, 24]. Our thinking about power is informed by ‘three dimensions of power’ work by Steven Lukes (1974) [25], and subsequent expansion by Veneklasen et al. (2002) to identify three main forms – visible, hidden and invisible [3]. A more comprehensive analysis of power within priority-setting following devolution is published elsewhere [26].

Intersectionality theory provides a structured way to engage with the complexity of social dimensions experienced and how these influence health, health decision-making and use of services, in light of devolution [27]. Social norms and forces (which include patriarchy and capitalism, legacies of colonisation such as tribalism, along with discriminations such as sexism, classism and ableism, among others) are useful to consider when examining the effects of policy decisions, such as devolution, on a range of citizens, with varying levels of (dis)advantage within society.

### Methods, participants and process

Interviews with 269 individuals and 14 focus group discussions with an additional 146 participants, were conducted in total between March 2015 – April 2016 (see Table 2). Fourteen national level key informants were selected purposively using a snowball approach, to identify other potential respondents who could contribute valuable information relating to priority-setting for health following devolution. National, county and some health worker level interviews were carried out by the first author (a non-Kenyan national, trained qualitative researcher) in English (RM). One hundred twenty county level decision-makers were interviewed across the ten diverse counties sampled for the study. These ten counties were selected to include representation of a range of poverty levels, geographic settings, cultural and social demography and health service coverage levels within the country. This has been described more extensively elsewhere (see Methods section and table 3 [18]). County level decision-makers were selected purposively to include politicians involved with decision-making for health, county treasury staff involved with budget guidance, gender and children’s office representatives and technical decision makers for health including members of the county health management team (see Methods section [18]). Interviews with 49 health workers from sub-county, health facility and community levels were carried out in three of the ten counties, selected to include counties which aligned with REACHOUT consortium^1^ data collection and which included representation for urban, rural agrarian and rural pastoralist settings. Eighty-six interviews with close-to-community (CTC) providers, their supervisors and community members and 14 focus group discussions were carried out with community members from two out of the three counties. This data was collected by Kenyan national researchers, trained in qualitative research as part of an ongoing REACHOUT CTC provider quality improvement study in two counties (urban and rural agrarian). We used topic guides to explore the priority-setting process for health following devolution, participants understanding of ‘health equity’, the factors which contribute to a person’s vulnerability including their ability to access or use health services and any changes to these factors since devolution. The topic guides were developed through an iterative process following informal discussions with national key informants, discussion between colleagues and a period of reflection and revision after data collection in one county to ensure questions elicited relevant responses.

**Table 2:** Respondent demographics

	Male	Female	# respondents
National key informant interviews			
County representative for county executive committee forum at national level	1	0	1
National Ministry of health	6	1	7
NGO/research institute/ donor	4	2	6
Total national respondents	11	3	14
County level in-depth interviews			
County executive committee member for health	6	3	9
Chief officer for health	7	3	10
Director/deputy director for health	17	2	19
CHMT member	19	13	32
Total county level health respondents	49	21	70
Children’s office representative	7	3	10
Gender representative	6	4	10
Member of county assembly (or representative)	15	5	20^a^
County treasury representative	6	0	6
Other county informants	3	1	4
Total county level non-health respondents	37	13	50
Multi-level in depth interviews			
Community health extension worker/ community health volunteer	6	6	12
Health facility in-charge	8	9	17
Hospital in-charge	6	0	6
NGO coordinator based at county level	1	0	1
Sub-county community health focal person	5	2	7
Sub-county medical officer	5	1	6
Total multi-level respondents	32	17	49
Community health in-depth interviews in two counties			
Community health volunteer	12	12	24
Community health extension worker	4	2	7^b^
Community health committee member	8	6	14
CHV team leader	4	9	13
Health facility in-charge	4	3	7
Sub-county community health focal person	3	1	4
Community key informants	11	6	17
Total IDI respondents	46	39	86
Photovoice participatory research in one county			
Youth	4	5	9
Total participants	179	98	278^b^
Community member FGDs in two counties			
FGDs	7	7	14

^a^Joint interview with 3 men and 1 woman

^b^Unrecorded gender one respondent

Maintaining a focus on social, structural and environmental inequities, photovoice participatory research was used to generate knowledge from nine young people (five female and four male), aged 16–18 years, who had dropped out of formal education in Korogocho informal settlement in Nairobi County. The young people were identified in collaboration between the research team and local community leaders. The process was used as a platform to enable the young people to record and reflect on their community’s strengths and on their own concerns through photography, to promote critical dialogue and knowledge and to increase their involvement in local decision-making by identifying responsive actions to be taken within their community [28]. We held six full-day sessions with participants during which we carried out initial introduction to photovoice, discussed group confidentiality, how to use cameras and consent. We also introduced the topic of health and discussed with young people the issues they wanted to explore relating to health in their community through their photos. In accordance with the participatory nature of photovoice research, participants decided to expand issues to also include life hazards, due to the strong linkages between life hazards and health. During the sessions, the young people went to the community to take photos and then identified photos for discussion, these were then printed and discussed together during the next session. This discussion included questions commonly used during photovoice studies, such as ‘Describe your photo? What is happening in the photo? Why did you take this photo? How does this affect us? What can we do about it?’ [29]. It provided a range of additional insights and the opportunity to learn more from those not typically included in health priority-setting, in keeping with the ‘diverse knowledge’ principle applied within intersectionality based policy analysis [23].

### Analytical process

We adopted a framework approach to analysis in order to classify and organise data according to the key themes, concepts and emerging categories [30]. This included an inductive aspect, which allowed meaning to emerge from the data through familiarisation with the data by reading and re-reading through transcripts [31]. Following this a thematic framework was developed, which drew on understanding of the literature, the objectives of the interview, the themes within the data collection tool and issues raised by the respondents themselves during interviews. Nvivo 10 software was utilised to manage and code data. Data coding was carried out by one researcher. Following coding, data was charted in order to summarise findings while still retaining its context and essence, based on data from all ten counties and enabling analysis of the similarities and differences of views and experiences from different participants with diverse social positionalities and operating at different levels of the health system [30] (see Table 1). The analysis and identification of themes was informed by the intersectionality wheel [8].

### Quality assurance and ethical considerations

Qualitative data was recorded with participant’s consent and transcribed verbatim. Data collection continued until saturation was reached and data was triangulated between sources to minimise bias. We reflected on our position as UK and Kenyan researchers and adopted reflexivity and positionality lenses within the analysis approach [23]. Being part of an embedded Kenyan institution (LVCT Health)^2^ and regular discussions and presentations with colleagues and other researchers within and outside Kenya were an important part of incorporating multiple perspectives in order to enhance rigour throughout the research process. Community and some health facility level respondents were interviewed by trained research assistants in Kiswahili or Kamba (depending on respondents’ preference). These interviews and discussions were translated to English, with a selection back-translated for quality checking. All participants were provided with information about the study and participants aged over 18 years gave informed written consent. Young people aged 16–18 years gave informed written assent, while informed written consent was sought from a parent or guardian. In addition to the usual information provided (about the study, use of information, freedom to withdraw without consequence) consent for the photovoice study also included permission for the research team to use a selection of photographs taken by the young people. Ethical approval was received from the relevant research ethics committees.

## Findings

**Fig. 2:**
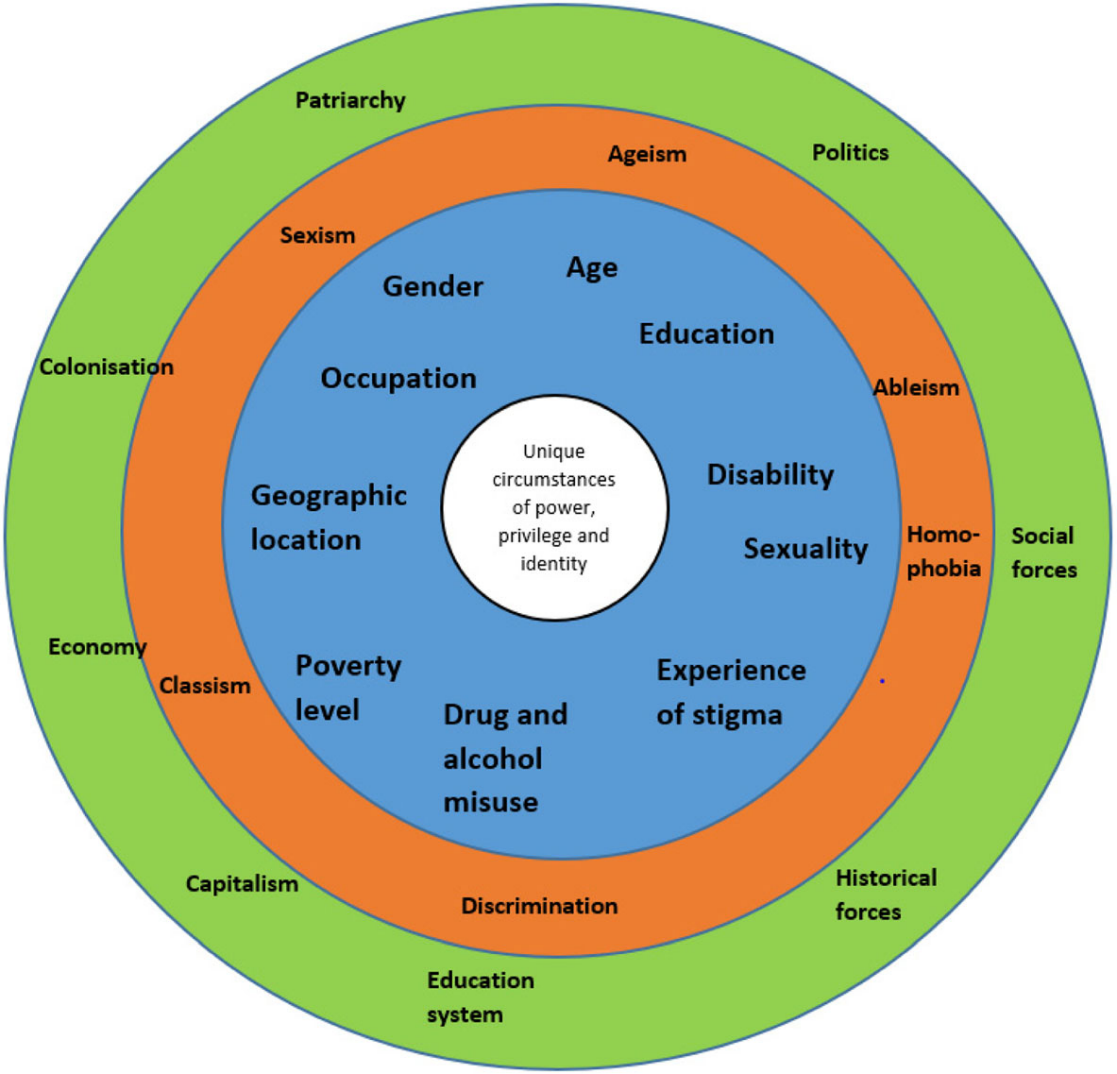
Intersectionality wheel for forces and structures, discriminations and dimensions of social inequality which interact to reinforce exclusion which emerged from our analysis, adapted from Simpson [8]

We present findings generated through the broader study conducted across ten counties, using the photovoice sub-study to bring to light examples of how social dimensions intersect and influence health and use of health services in the lives of individuals, as described and captured through photography by nine young people living in an informal settlement. Respondents described a range of social dimensions which we observed to occur in clusters, intersecting and contributing towards a person’s experience of risk and/or ill health (see Fig. 2, Simpson’s intersectionality wheel adapted to the findings from this study). The intersection of two or more dimensions shapes the level of power and privilege held by that person. We found this was influenced by larger social forces, structures and discriminations at work within the context. The following section will highlight findings for the four most commonly discussed dimensions (age, gender, geographic location and poverty) and how they converge to influence vulnerability.

### Age

**Fig. 3:**
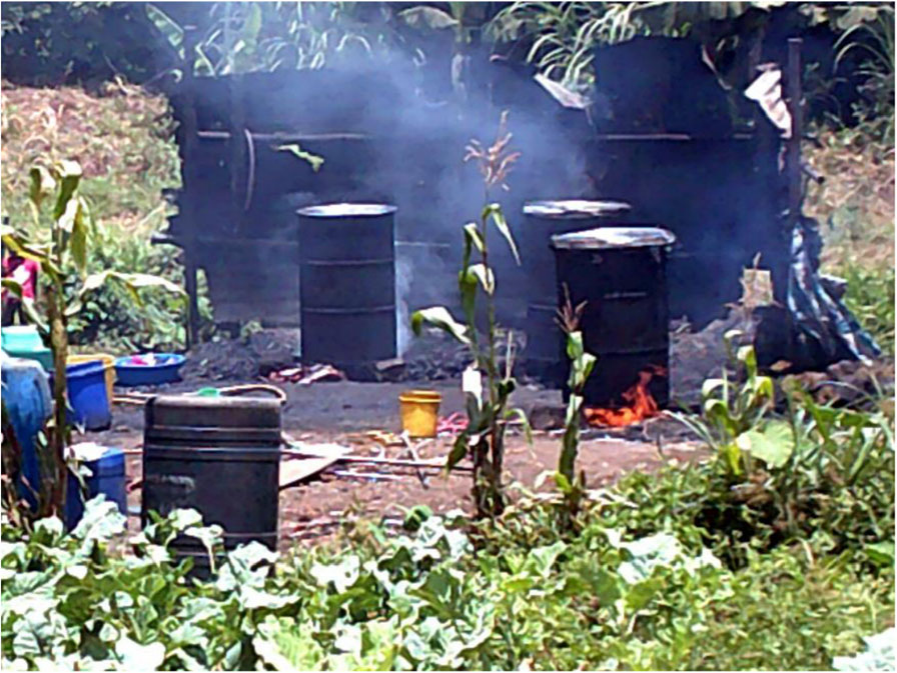
Chang’aa brewing, Photographer Irene Akoth

Age was identified as a social dimension which could influence a person’s power and contribute to vulnerability and challenges using health services across the life cycle. Children, young people and the elderly were most commonly identified as being potentially vulnerable, by all types of respondent across the health system and within the community. This was particularly when age intersected with other categories, such as poverty, lack of social capital, being an orphan, or neglected (such as children with alcoholic parents who did not provide the care and love needed, linked with Fig. 3). Children who were orphaned, were felt to have lack of power, as a consequence of their age and lack of social capital. This often converged with poverty and limited opportunity for education, which limited their ability to raise enough money to meet their basic needs, potentially resulting in neglect of their health, as described by county respondents and youth photographers.

“*It’s also in the slums that you’ll get child-headed families. Yes, parents have died, but the older child assumes the responsibility of the parents. Yeah and also, so health may not be really a priority, why? Because that child is busy thinking about how to fend for themselves. So will he or she go to hospital when they are unwell when they can go and do some job somewhere?”* County Non-Health Respondent, Female15

**Fig. 4:**
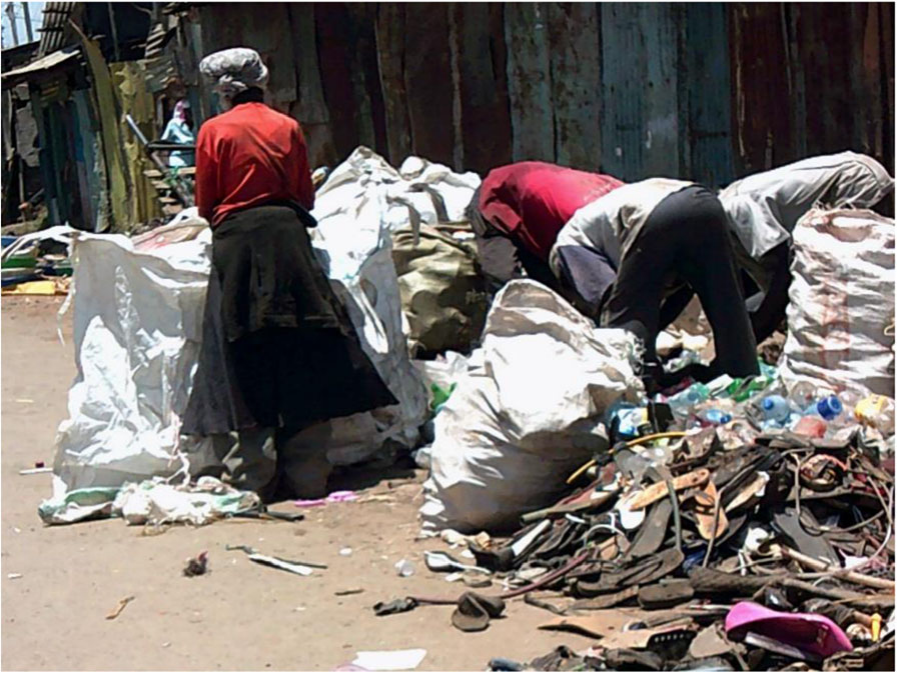
Searching for plastic to recycle, photographer Rhonda Namwendwa

Youth photographers highlighted that age, limited social capital, poverty, and limited education can converge with location when orphans and other vulnerable children live within an informal settlement, resulting in these children and youth experiencing limited employment opportunities; this lack of power to choose their livelihood leads to these children being obliged to so scavenge for plastics to recycle in order to earn an income for the family (see Fig. 4).

Alcohol and drug misuse among youth was commonly discussed, by community members and CHVs in the urban county, as intersecting dimensions which contribute to vulnerability. Youth photographers acknowledged the challenges associated with drug and alcohol misuse among their age mates, relating it to peer pressure and unemployment. It was felt to contribute to loss of power through intoxication and was felt to converge with gender and poverty, potentially increasing a girl’s exposure to sexual violence, risky sexual practices, and feeding into a cycle of poverty (see Fig. 3).

*“Chang’a makes parents forget their duties. The things that they do are harmful to our health. If someone gets used to those drugs he becomes idle. The people who cook and sell these things are in danger, if she is a girl she may be raped.”* Caption by a youth photographer.

According to policy, representatives for youth (and women and people with disabilities) should be invited and given opportunity to participate during community meetings for decision-making. None of the youth photographers described having participated in these meetings (although this was not explicitly probed). Due to the lack of ‘youth friendly’ services, youth did not attend for health education or for services at the health facility as frequently as needed, increasing their vulnerability, with girls and young women in particular at risk of teenage pregnancy, due to a lack of the needed health information and the need for youth specific sexual health education. Youth photographers expressed appreciation for the work which CHVs carry out and highlighted how they encourage attendance for health services.

*“My age mates really don’t value visiting health centres, yet they are the most with early pregnancy issues so they (CHWs) usually advise on the importance of visiting hospitals.” Youth photographer film*

Other age groups were also considered potentially vulnerable due to changing social capital and geographic location. For example, some respondents identified that some elderly people who live in rural areas are vulnerable, since their children have moved to larger cities and do not return home regularly to provide support or care. Other dimensions can converge with advanced age to increase the challenges experienced in using health services, including geographic location, poverty level or disability. As a result, elderly persons who live far from a facility, who have limited mobility and who are too poor to afford the costs for transport were described as experiencing challenges in accessing facility-level health services.

“*So we leave our elderly people back in the villages, and that’s a very vulnerable group.”* County Non-Health Respondent, Male02

While the Constitution identifies that youth should be involved with decision-making, there was little other discussion about practical examples for how people from other ‘vulnerable’ age groups can participate in decision-making for health or benefit from improved access to health services following devolution.

### Gender

County level respondents recognised that women experience a range of risks, which they identified as a combination of both biological and social. The biological risks described were those associated with pregnancy and childbirth occurring as a result of women’s physiology, while social risks were those occurring as a consequence of gendered power imbalances between men and women, boys and girls. These may intersect with other factors such as her age, tribe and geographic location, and by the strength of patriarchal norms. Depending on the dimensions present in a woman’s life at a certain point in time, this was described as potentially leading to the occurrence of dangerous practices such as child marriage, female genital mutilation (FGM) and gender-based violence (GBV). Gendered and patriarchal social norms led to limited power among women, due to their inability to own land, to control finances or make decisions about seeking health care or family planning, without first gaining her husband’s permission. This was most commonly described in pastoralist counties.

**Fig. 5:**
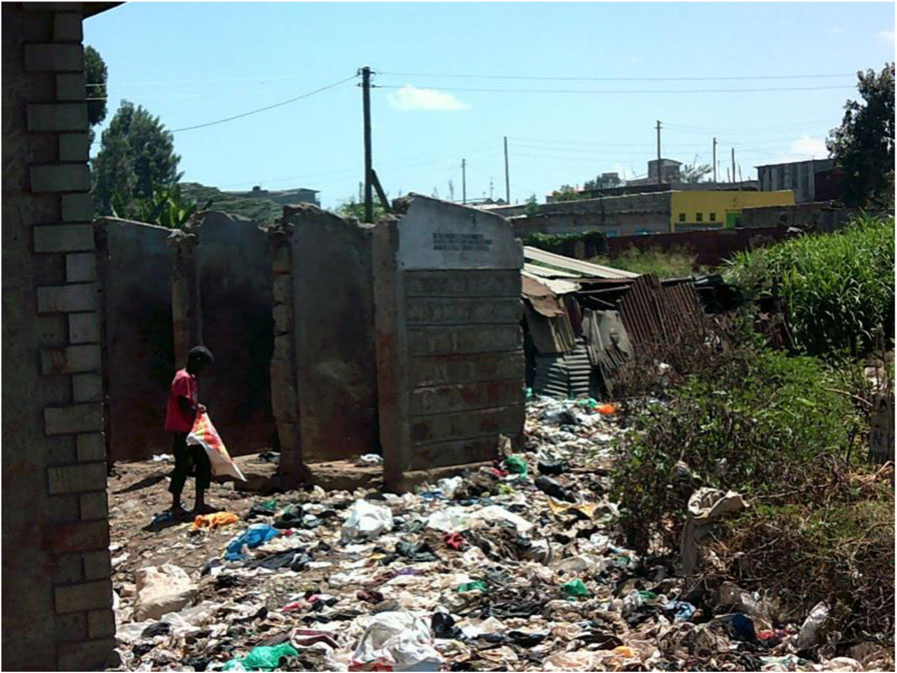
Toilets without doors identified as high risk location for rape, Photographer Verine Adhiambo

Gender-based violence (GBV) was described in many counties. Gender was felt to intersect with geographic location and age, with adolescent girls living in informal settlements seen as at greatest risk of exposure to GBV, although other women were also identified as at risk (see Fig. 5). Solutions identified in response by youth photographers were renovating toilets with a door and building toilets within housing plots.

*“This is a toilet. I don’t like the way it looks [because] it’s built outside a plot (group of low cost different adjacent houses owned by one landlord and usually sharing the same roof and/or with a common wall connecting the houses). It doesn’t make sense. A girl can easily get raped while going to the toilet.”* Youth photographer FGD

While women were identified as being more vulnerable compared with men, there were different vulnerabilities described for men, particularly by respondents at community level. There was a perception described by men that health services are primarily intended for women and children rather than men, which created a barrier to them seeking help when needed. Meanwhile within Nairobi drug and gang-related violence among young men was highlighted.

Since devolution, policies specify that representatives for women should be invited to participate during public meetings in order to address some of the historic, social and political norms and forces, e.g. patriarchy, which have contributed to their historic under-representation within decision-making platforms. In some counties, particularly pastoralist ones, where patriarchal social norms are particularly strong, unequal power relations and sexist discriminations persist, working to hinder women’s active participation in decision-making.

*“So they [women] are hardly fully represented in those public participation things …they miss out of the quorums and number two even if they come up they may not be able to speak out.”* County Non-Health Respondent, Female25

**Fig. 6:**
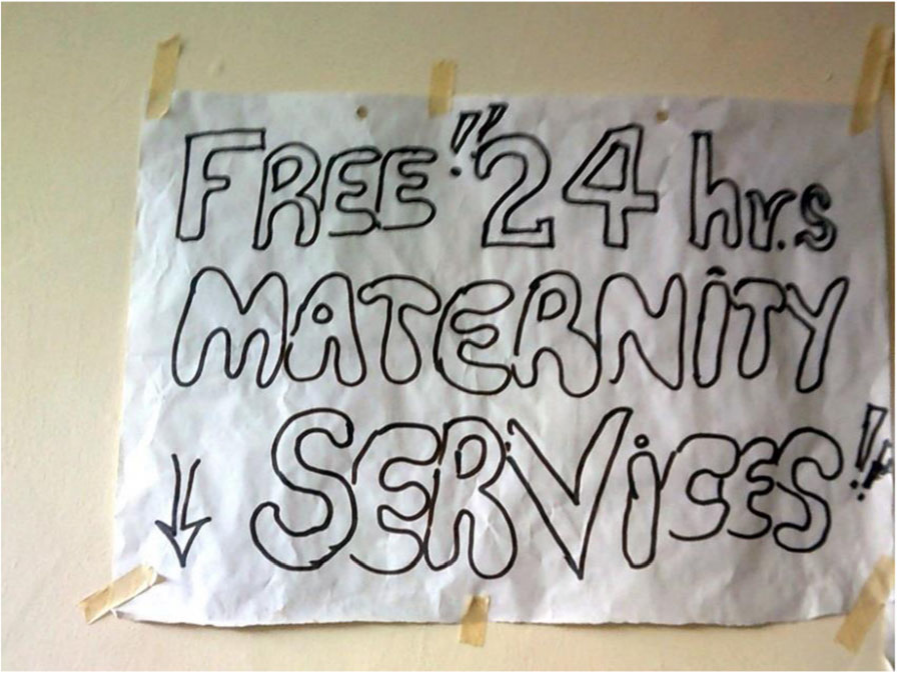
Sign advertising free maternity services at newly built government health facility in an informal settlement, Photographer Mary Wanjiku

Poor women living in hard-to-reach areas (in a remote community or an informal settlement) may struggle to access and use services, such as skilled delivery, due to lack of funds for transport. This in turn may lead to inequitable morbidity and mortality. This was influenced by the extent to which the health system was designed to extend services (or not) to women who experience multiple layers of disadvantage. Respondents from many counties highlighted that one of the main priorities following the introduction of devolution reforms was maternal health, with many county governments having built new health facilities, often in hard-to-reach areas or in informal settlements (see Fig. 6).

### Geographic location

Geographic location was perceived to influence a person’s power and health through exposure to environmental risk, their (in)ability to access health services and to participate in decision-making to set priorities. Exposure to environmental risk was most commonly discussed in urban areas^3^, while geographic access to services was most frequently raised in pastoralist and agrarian counties. The environmental risks included: inadequate sanitation, which varied between villages within the informal settlement; spread of tuberculosis in poorly ventilated thatched houses in rural areas; wound infections from working in dirty water in urban informal settlements; diarrhoea and malnutrition as a consequence of lack of clean water and sanitation; exposure to raw sewage, pollutants or toxic fumes from burning rubbish.

*“Those people in dump sites they usually have many sicknesses, because of the working conditions they work in. Because you find every time it’s burning and when they continue to inhale that smoke they catch chest related sicknesses.”* Community Key Informant, Male11

**Fig. 7:**
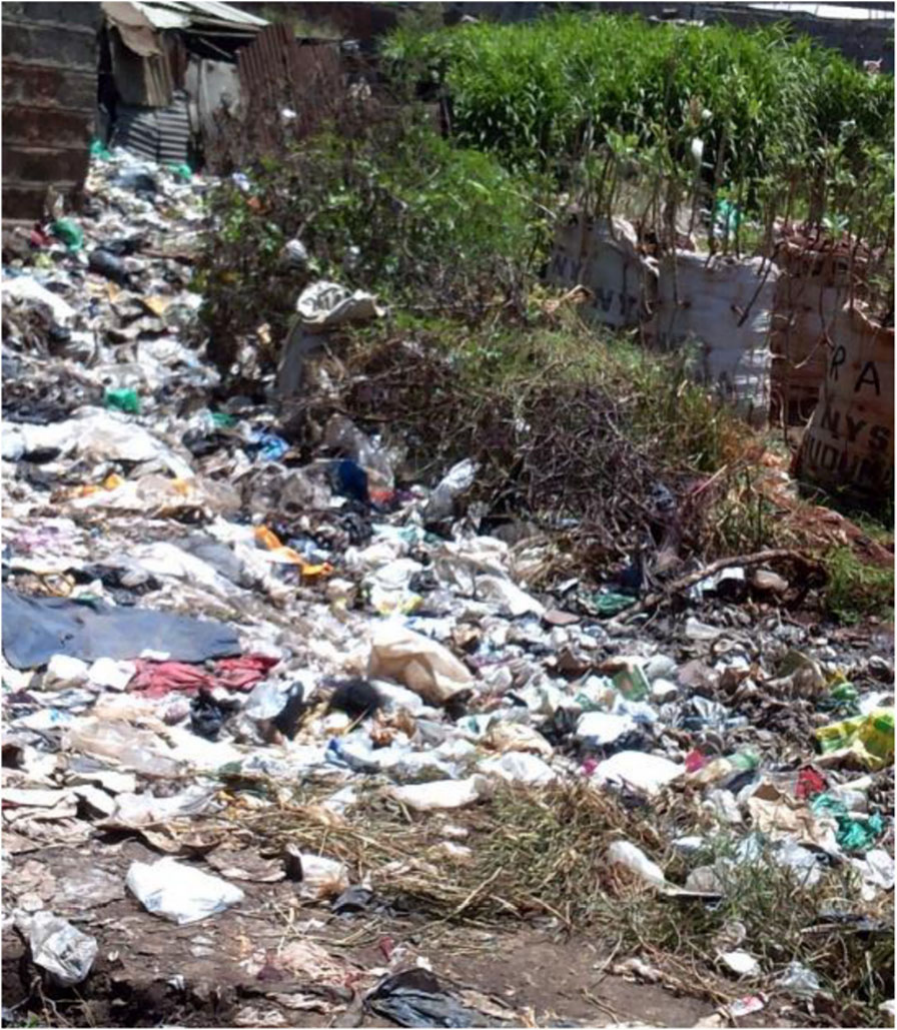
Rubbish discarded within Korogocho informal settlement. Photographer Adan Iya

**Fig. 8:**
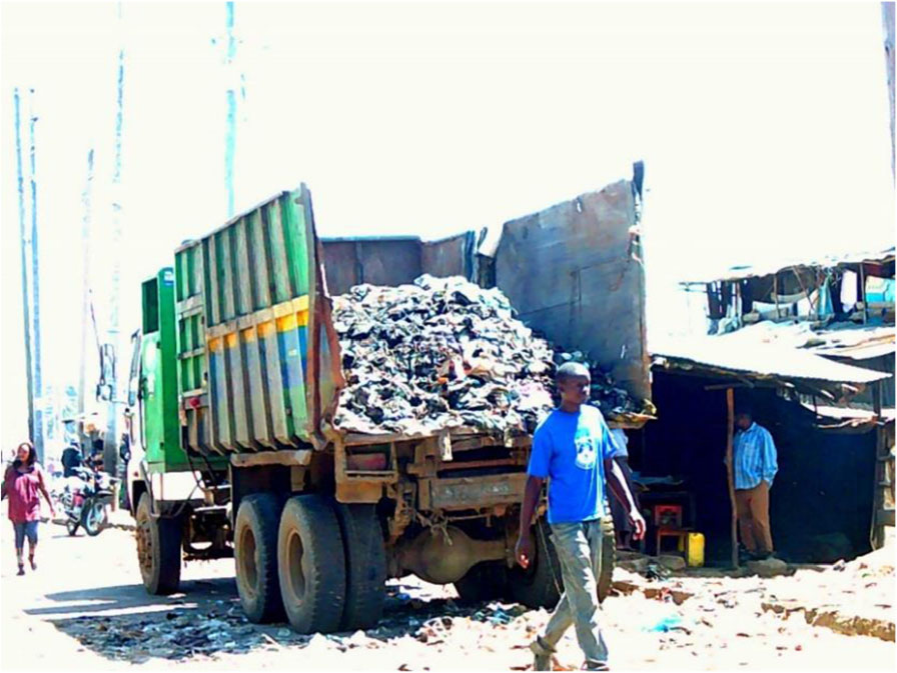
Lorry collecting rubbish. Photographer Rufus Njoroge

The rubbish abandoned in some villages within the informal settlements was highlighted by many of the youth photographers as a cause of ill health (see Fig. 7). They also highlighted the introduction of rubbish collection (see Fig. 8). This intervention was described as having been facilitated by the youth, demonstrating a degree of agency and power in addressing a risk factor for ill health. Participants did not describe any link between this improvement and changes brought about by the county government as a result of devolution.

*“A lorry that collects garbage… I loved it, because the people collecting the garbage are the youth.… [so] the community becomes clean.”* Youth photographer FGD

In fact, some respondents identified that despite devolution of decision-making providing the opportunity for counties to address the underlying causes of ill health, such as poor sanitation and clean water supply, these actions were not considered politically appealing. As a result, county governments prioritised visible interventions such as construction of hospitals. This has been described more fully elsewhere [18].

People living in the most remote areas, particularly in pastoralist counties, were described as experiencing the greatest geographic barriers to accessing and using health services. This is as a result of location converging with poverty to limit the most vulnerable people from using services due to the barrier created by inability to afford transport costs. This also creates a challenge for the same people to joining public participation meetings, which are typically held in main towns. In some cases, the county government have built health facilities in previously underserved areas. For those living in urban informal settlements and in certain areas in rural counties, insecurity creates a barrier which hindered use of services, particularly at night (regardless of the geographic availability of the service).

**Fig. 9:**
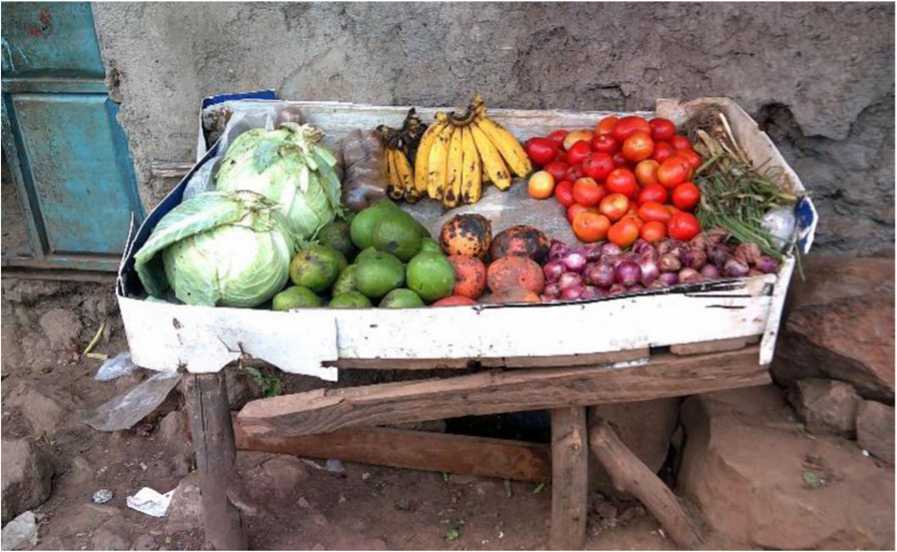
Fresh fruit and vegetables for sale in Korogocho informal settlement, Nairobi

Further evidence of the mutually constitutive dimensions of vulnerability was highlighted through the photovoice research, which revealed the variation in the level of poverty and exposure to risks, even within one informal settlement, with youth highlighting the differences within the same settlement. Congestion, unsafe construction, presence of alcohol abuse, and limited access to toilets are felt to contribute to risk. Meanwhile, access to private yard, location of a person’s home near a police station, maintenance of the environment and ability to generate additional food or income e.g. sack-based kitchen garden, presence of a cow or a shop (see Fig. 9), are felt to be protective factors which instil resilience to residents living in those areas. This variation demonstrates how ‘social location’ cannot be easily predicted by single factors such as geographic location.

*“It (selling fruit) is good because it improves our livelihood as people of Korogocho, through giving us a living and hence good lives.”* Youth photographer caption

### Poverty

Poverty was felt to commonly intersect with all other social dimensions, leading to limited power and lack of agency, with dimensions being mutually constitutive by intersecting to constitute a social location that is inseparable from them as distinct categories. People living in geographically marginalised areas often facing linked challenges of limited income generating options, and poor living and working conditions. This in turn may lead to increased exposure to environmental health risks, compared with people who are not poor.

Since devolution, the national government have removed user fees as a means to reducing the barrier presented by poverty to seeking health care which was felt to have increased uptake of services. Those who experience poverty and live in a remote location continue to experience challenges with attending the health facility and participating in public meetings, due to lack of funds to pay for transport needed to reach the facility or meeting (as described above). In addition, limited availability of drugs and supplies to health facilities means that they frequently experience drug stock-outs, thereby creating to barrier to people who are poor from receiving effective care.

*“When you get to the doctor, the tests are run on you and prescriptions given and maybe you have no money to buy the drugs; you went there knowing you will be given drugs and then you are referred to a chemist and you don’t have the money.”* Youth photographer focus group discussion

### Other social dimensions

Despite the increasing availability of primary health services in formerly underserved areas, citizens who are most marginalised still struggle to use these services, even when they are available. Many counties have not yet addressed the constraints to accessing services, such as the acceptability of skilled delivery by engaging with cultural and religious beliefs and community perception of health workers. By contrast, a minority of the counties studied have introduced demand generation strategies, such as community health approaches, where community health volunteers (CHVs) and traditional birth attendants (TBAs) encourage pregnant women to attend the health facility and have seen encouraging results, with increasing skilled delivery rates.

Stigma due to disability, HIV status or sexual orientation were less frequently discussed social dimensions. However, where these were discussed, respondents highlighted that these dimensions can create barriers to using available health services. Respondents from several counties highlighted the prevailing stigma towards people with disabilities, particularly people with mental illness. This stigma appeared to converge with location, and was most commonly described by respondents from more remote settings, where cultural beliefs associated mental illness with wrongdoing.

*“Another reason is the hiding of the persons living with disability, done by their family members, out of shame… and so they don’t get the health services they need.”* FGD Male01

Stigma towards gay and lesbian persons was also highlighted in several counties, along with the need to introduce policies which safeguard their interests. There were no examples shared, however, of any changes to policy or actions introduced following devolution.

*“So here we have actually quite a significant group of gays and lesbians. So you know those are vulnerable groups… given the stigma I think that is also quite high. So those are guys that we need to make sure that we create a very friendly system and even policies, so that we safeguard their interests.”* County Health Respondent, Male07

Many respondents across health systems levels and within the community described the vital role which CHVs can play to increase access to and the use of health services by everyone in the community.

“*I wanted to report that those who are disadvantaged are the ones given priority. They (CHVs) care for them the most.”* Community Key Informant, Male02

## Discussion

Our findings reveal how wide social forces such as patriarchy intersect with specific manifestations of disadvantage such as poverty, neglect, family breakdown and life stage (e.g. adolescence, childhood) in particular geographic contexts (e.g. urban informal settlements, remote rural areas). We found that power relations intersect to produce specific vulnerabilities for specific groups in specific contexts (e.g. adolescent girls and boys in urban informal settlements or pastoral women). In other instances, our findings reveal what kinds of intersecting inequities are perceived by key actors within the priority-setting process since devolution, identifying examples of the established insight (that inequities shape the social determinants of health), which are grounded in the tacit knowledge of these key decision-making stakeholders. Devolution presents an opportunity to transform power dynamics, relationships and social dimensions, by empowering communities and individuals to participate in setting priorities and using health services.

We found that despite policy recommendations for inclusion of marginalised people within decision-making processes [17], social power relations, including sexist, ageist, homophobic and ableist norms and hierarchies shape expectations, knowledge and capacities, thereby limiting opportunities for historically marginalised people present at these meetings to actually set agendas and influence decisions and outcomes. This is because the exercise of power is more subtle and complex than physically being present when decisions are made or consulted upon and has been described elsewhere [26]. ‘Invisible power’ remains in place, limiting access to information and failing to engage marginalised citizens to make informed choices or decisions [3, 25]. This is in keeping with previous studies which acknowledge the influence of social hierarchies, economic and political division on citizens’ participation within committees, which operate within existing hierarchies and patterns of power and privilege [32, 33].

We identify the main social dimensions (blue), discriminations (orange) and social forces (green) described by participants to create a Kenya specific version of the intersectionality wheel (see Fig. 2). The data focuses on the inner circle, with the words in bold indicating the key themes of the findings. Although participants didn’t often trace the intersecting social forces underpinning these, we have reflected upon this in the discussion section, due to the importance of retaining this focus on drivers of inequity. Through our study we identified some dimensions that strongly intersect to constitute particular ‘social locations’ shaping health opportunities - for example particular intersections of gender with age and geographic location with poverty – these are discussed below.

## Gender and age

Different social vulnerabilities emerge at different life stages through intersection with other social forces. At community level, societal and cultural norms, such as patriarchy and discriminations such as sexism, continue to disempower women and girls, contributing to FGM, early marriage, low education and low economic empowerment (see Fig. 2). This perpetuates a cycle where particularly disadvantaged women and girls are at increased risk of complications during delivery, as a result of scarring from FGM [34] and teenage pregnancy as a result of early marriage. A recent study following devolution in Kenya, describes the convergence of gender with disability and poverty, in hindering disabled women from accessing skilled delivery, despite the presence of the free maternity programme [20]. Gender-based violence was commonly discussed by photovoice participants, who were adolescents who had dropped out from full-time education, living in an informal settlement with high levels of poverty and alcohol misuse in the surrounding community, in keeping with risk factors for GBV as identified through previous studies in Kenya and globally [35, 36]. ‘Invisible power’ is exercised by the powerful in society to shape the aspirations, expectations and desires of the least powerful. Lack of a sense of agency and fatalism are the result of the exercise of hidden power which shapes what informal settlement dwellers may aspire to, in keeping with an earlier study in informal settlements in Nairobi [37]. Within our study this was highlighted by the youth’s solutions to the high levels of gender-based violence within their community, which centred around treating the ‘symptoms’ (such missing toilet doors leading to risk of rape), rather than addressing the underlying causes (such as inequitable gender relations and lack of effective governance).

## Poverty and geographic location

Poverty and geographic contexts contribute to differing health risks and ability to use services, due to lack of transportation and exposure to environmental exposures, such as pollution [38]. Scarce and inconsistent employment opportunities within informal settlements compounds poverty [39], limiting choice and power for those seeking work and blocking the pathway out of poverty provided by regular meaningful employment [38]. Geographic location may intersect with other dimensions, such as poverty, limited education and age to further limit employment opportunities, which can lead residents to undertake jobs with negative health implications. This is in keeping with findings from an intersectionality analysis of violence in Mexico, where lack of alternative opportunities led men (in this context gendered expectations meant that men were obligated to provide financially for their families), to take jobs which placed them at increased risk of exposure to violence [40]. As photovoice respondents highlighted, living in an informal settlement did not result in equal exposure to risk factors for ill health. Myriad variation between and within villages, even within one informal settlement, led to varied exposure to risk factors, highlighting how multiple dimensions converge within the lives of residents, leading to variations in unique social ‘location’ and thus levels of vulnerability and resilience.

Priority-setting decisions influence the availability (or lack) of health services to communities according to geographic location. Prior to devolution, historic forces including colonisation had contributed to wide variations in health service coverage, with those living in formerly marginalised areas having limited essential health service coverage [11]. Since devolution, many county governments have sought to address this by increasing availability of primary health services in previously underserved areas. Similar to other countries [41, 42], devolved counties have generally been slow to address barriers to service use relating to cultural and religious beliefs, women’s autonomy and access to knowledge and information about health and services. As a consequence, in some pastoralist counties women continue to deliver at home, despite investment in new health facilities by county government, as highlighted previously [43]. Meanwhile supply chain gaps, a common challenge following decentralisation [44], has resulted in those who are most poor remaining excluded from effective treatment and care, despite the removal of user fees and introduction of free maternal health policy.

## Implications for policy and practice

Power relations at county, community and household level shape health, including the processes and impacts of devolution. Transformation of the social determinants of health requires challenging the status quo. Actors in positions of authority are unlikely to actively seek to transform the existing power relations from which they benefit. This means that negative social relations and practices, such as patronage and nepotism, can easily embed after health reforms, potentially leading to ‘capture’ of funds and priorities by more ‘politically influential’ individuals and groups, with continued neglect of historically marginalised groups [45]. Any efforts towards transformation will often bring resistance from those who benefit from the current balance of power [46]. In response to the current power relations, which we have sought to describe through this paper and elsewhere [26], there is need to facilitate processes of empowerment for less powerful actors in the political process of priority-setting in devolved settings. ‘Making visible’ the key inequities, by using the tacit knowledge of actors at this level may be an important step in this process of social change, as it may identify ‘groups’ who need support/capacity building and the importance of being sensitive to multiple power relations at play within these groups. Devolution as a process will need to include such support and capacity building, otherwise existing power relations may become entrenched, rather than transformed for the benefit of people who are currently vulnerable/marginalised [45].

We propose that counties take up the opportunity devolution presents for introducing intersectoral approaches, with collaboration with other departments within the county and strong social participation. These approaches can challenge the existing balance of power, by introducing actions which seek to transform the social determinants of health and reduce citizens exposure to risks for ill health, particularly among ‘vulnerable groups’, learning from examples of this in Latin America [47]. Community-based primary health care provides a potential platform for delivery of this intersectoral approach, with CHWs acting to both extend services and create change in social determinants within their communities [48]. First however, they themselves must be empowered, since they live, work and experience the same social norms and discriminations as the citizens within their community [49, 50]. They will need the support of both their community and the health system in order to perform this unique role [51].

## Progression of intersectionality practice

Our study adds to the literature a consideration of how health vulnerability is shaped by power inequities, *including voice in the process of priority setting,* so that this needs to be built into change processes. Applying an intersectionality lens following health system reforms, such as devolution, provides the scope to consider implications for ‘marginalised’ groups. Use of intersectionality approaches within public health have to date focussed on the experiences of select groups, such as HIV positive people with disabilities, HIV positive men who have sex with men, HIV positive men [52–54]. Through this study we have sought to take forward intersectionality practice by applying its principles (see Table 1) to understand how power relations intersect to create a range of health vulnerabilities across the general population, according to stakeholders, following health systems reforms.

## Limitations

The diversity between Kenya’s 47 counties may limit generalizability of findings. We aimed to mitigate this by purposively selecting ten study counties to reflect demographic, geographic, social, cultural, economic and health service coverage differences. Research was carried out across health systems and community levels in keeping with intersectionality multiple levels of analysis principles. Interviews were conducted with county leaders across ten counties, but with health workers in three, community members in two counties and with young people in one county, due to time and resource constraints. We have sought to ensure breadth across counties and types of respondents through our study, however, there is need for future research needs to look specifically at how intersectional power relations play out in a specific context in more depth to inform concrete change processes within that location. Reforms under devolution and power relations within priority-setting and at community level form part of a complex adaptive system, driven and influenced by the political economy. Our research presents a snapshot of this at a particular time and place. However, we have tried to consider how historical trajectories have influenced the process and impacts of devolution. Positionality of the main interviewer as a foreign researcher may have may have inhibited some respondents from speaking freely or influenced the way they framed their views. Conversely, some respondents may have felt less threatened and discussed more due to the interviewer’s ‘outsider’ status. We have sought to reflexively recognise how our position as researchers influenced the study design and analysis.

## Conclusions

Devolution presents opportunity for progress towards universal health coverage. Achieving this aim requires county authorities to address the social determinants of health, by transforming the social forces, structures and discriminations that maintain power imbalances and inequities. We find that use of an intersectionality lens following introduction of reforms, enables opportunity for holistic scrutiny of the potential for devolution to increase or reduce vulnerability and marginalisation for people who are disadvantaged by existing power relations. Intersectional analysis thus provides a useful approach for analysing health reforms and we recommend their continued and further application within health systems research to inform change processes.

## Footnotes

1 REACHOUT is an ambitious five year international research consortium aiming to generate knowledge to strengthen the performance of CHWs and other close-to-community (CTC) providers in promotional, preventive and curative primary health services in six low- and middle-income countries in rural and urban areas in Africa and Asia, including Kenya.

2 LVCT Health is a national Kenyan non-governmental organisation with experience in HIV service delivery and HIV and community health systems research.

3 This may have been as a consequence of the insights provided through photovoice research (only carried out in Nairobi).
